# CD36 Deficiency Inhibits Retinal Inflammation and Retinal Degeneration in *Cx3cr1* Knockout Mice

**DOI:** 10.3389/fimmu.2019.03032

**Published:** 2020-01-08

**Authors:** Sophie Lavalette, Jean-Baptiste Conart, Sara Touhami, Christophe Roubeix, Marianne Houssier, Sébastien Augustin, William Raoul, Christophe Combadière, Maria Febbraio, Huy Ong, Sylvain Chemtob, José-Alain Sahel, Cécile Delarasse, Xavier Guillonneau, Florian Sennlaub

**Affiliations:** ^1^Institut de la Vision, Sorbonne Université, INSERM, CNRS, Paris, France; ^2^Université de Tours, Inserm, N2C UMR 1069, Faculté de Médecine, Tours, France; ^3^Sorbonne Université, Inserm, CNRS, Centre d'Immunologie et des Maladies Infectieuses, Cimi-Paris, Paris, France; ^4^Department of Dentistry, University of Alberta, Edmonton, AB, Canada; ^5^Faculty of Pharmacy, Université de Montréal, Montreal, QC, Canada; ^6^Departments of Pediatrics, Ophthalmology and Pharmacology, Université de Montréal, Montreal, QC, Canada

**Keywords:** age related macular degeneration, mononuclear phagocyte, CX3CR1, CD36, IL-6, photoreceptor

## Abstract

**Background:** CD36, a member of the class B scavenger receptor family, participates in Toll-like receptor signaling on mononuclear phagocytes (MP) and can promote sterile pathogenic inflammation. We here analyzed the effect of CD36 deficiency on retinal inflammation and photoreceptor degeneration, the hallmarks of age-related macular degeneration (AMD), that characterize *Cx3cr1*^−/−^mice.

**Methods:** We analyzed subretinal MP accumulation, and cone- and rod-degeneration in light-challenged and aged, CD36 competent or deficient, hyper-inflammatory *Cx3cr1*^−/−^ mice, using histology and immune-stained retinal flatmounts. Monocytes (Mo) were subretinally adoptively transferred to evaluate their elimination rate from the subretinal space and Interleukin 6 (IL-6) secretion from cultured Mo-derived cells (MdCs) of the different mouse strains were analyzed.

**Results:** CD36 deficient *Cx3cr1*^−/−^ mice were protected against age- and light-induced subretinal inflammation and associated cone and rod degeneration. CD36 deficiency in *Cx3cr1*^−/−^ MPs inhibited their prolonged survival in the immune-suppressive subretinal space and reduced the exaggerated IL-6 secretion observed in *Cx3cr1*^−/−^ MPs that we previously showed leads to increased subretinal MP survival.

**Conclusion:** *Cd36* deficiency significantly protected hyperinflammatory *Cx3cr1*^−/−^ mice against subretinal MP accumulation and associated photoreceptor degeneration. The observed CD36-dependent induction of pro-inflammatory IL-6 might be at least partially responsible for the prolonged MP survival in the immune-suppressive environment and its pathological consequences on photoreceptor homeostasis.

## Introduction

Age-related Macular Degeneration (AMD) is a common (1), highly heritable, neuroinflammatory disorder characterized by central, sizeable deposits under the retinal pigment epithelium (drusen) in its early form and choroidal neovascularisation (CNV, wet AMD) or an extending lesion of the outer central retina (geographic atrophy) in its late form (2).

We (3–6) and others (7, 8) showed that both advanced forms [wet AMD (5) and Geographic Atrophy, GA (3, 4, 6)] are associated with non-resolving accumulation of mononuclear phagocytes (MP) in the subretinal space. MPs are a family of cells that include monocyte (Mo), resident macrophages (rMϕ) such as microglial cells (MC), and monocyte-derived inflammatory macrophages/dendritic cells (MdC) that arise during inflammation (9). Physiologically, the subretinal space does not contain blood- and lymphatic-vessels, and is additionally devoid of immune cells, including resident MCs (4, 5, 7, 8, 10). Among others this particularity is due to the potent immunosuppressive, pro-apoptotic factors produced by the RPE that eliminate infiltrating leukocytes (4, 11). While MP accumulation observed around large drusen in early AMD (4, 6) might control debris accumulation, we showed that MdCs invariably participate in the infiltrate in and around GA lesions (6), where they are closely associated with rod and cone degeneration (3). Indeed, MPs, and in particular MdCs, an important source of inflammatory cytokines that play a critical role in animal models of pathological choroidal neovascularisation (CNV) (12, 13) and photoreceptor degeneration (5, 6, 14–18). In particular we showed that the inflammatory cytokines CCL2, IL-6, and IL-1 lead to the excessive MdC accumulation, diminish the subretinal immunosuppressivity and induce photoreceptor degeneration and CNV, respectively (3, 4, 6, 18, 19).

In the eye, CX3CL1 is constitutively expressed as a transmembrane protein in inner retinal neurons (20, 21) and provides a tonic inhibitory signal to CX3CR1 bearing retinal MCs that keeps these cells in a quiescent surveillance mode under physiological conditions (5, 22). *Cx3cr1* deficiency in mice leads to a strong increase of subretinal MP accumulation with age, after light-challenge and laser-injury (5, 23, 24), in diabetes (25), and in a paraquat-induced retinopathy model (26).

We showed that *Cx3cr1*-deficient MPs express high levels of Apolipoprotein E, also observed in humanized transgenic mice expressing the AMD-risk APOE2 isoform (TRE2-mice) and subretinal MPs of AMD patients (4, 6, 27). The excessive expression of APOE in turn can activate the CD14/TLR2/TLR4-dependent innate immunity receptor complex (IIRC) on MPs (4, 27), likely by modifying the cholesterol content of the lipid raft, in which the complex is located (28). Its activation in subretinal MPs induces CCL2 (27), which increases Mo recruitment (6), but also IL-6, which we show reduces RPE FasL expression and MP elimination (4). The age-dependent accumulation of subretinal *Cx3cr1*-deficient MPs is associated with a significant degeneration of rods and cones (5, 29, 30), but not with RPE atrophy. They therefore quite accurately model the transitional zone of GA and a subtype of GA called incomplete outer retinal atrophy [iORA; (31)].

CD36, a member of the class B scavenger receptor family, is expressed in a variety of cell types and binds a diverse array of ligands (32). It is expressed on Mϕ, MC, RPE cells, and microvascular endothelial cells (32), all cell types potentially involved in AMD. On vascular endothelial cells CD36 has been shown to mediate the antiproliferative effect of the thrombospondins (33, 34). Its expression on RPE cells has been shown to influence the phagocytosis of rod outer segments (ROS) (35, 36), in particular oxydized ROS (37, 38), but also oxydized lipoproteins (39), whose CD36-mediated uptake activates the NLRP3 inflammasome in the RPE (40). Finally, it is expressed on MC and Mϕ where it is a co-receptor of the toll-like receptor 2 (TLR2) and activates a proinflammatory signaling cascade and the release of inflammatory cytokines (41, 42). On the other hand, internalization of apoptotic bodies via CD36 can inhibit proinflammatory pathways (43, 44).

We have previously shown that CD36 expression regulates COX-2 and VEGF expression in RPE cells. Its deficiency leads to a slow, age-related choroidal involution and mild retinal degeneration (45). On the other hand deficiency of CD36 protected against subretinal inflammation and photoreceptor degeneration in an acute model of light toxicity, as it inhibited the release of neurotoxic IL-1beta from subretinal MPs (42).

Here we investigated the influence of CD36 on inflammation and photoreceptor degeneration in *Cx3cr1*-deficient mice that we previously showed develop pathogenic subretinal MP accumulation with age and after a non-toxic light-challenge.

## Results

### CD36 Deficiency Prevents Age-Related, Chronic, Pathogenic Subretinal Inflammation in Cx3cr1-Knockout Mice

**Figure 1:**
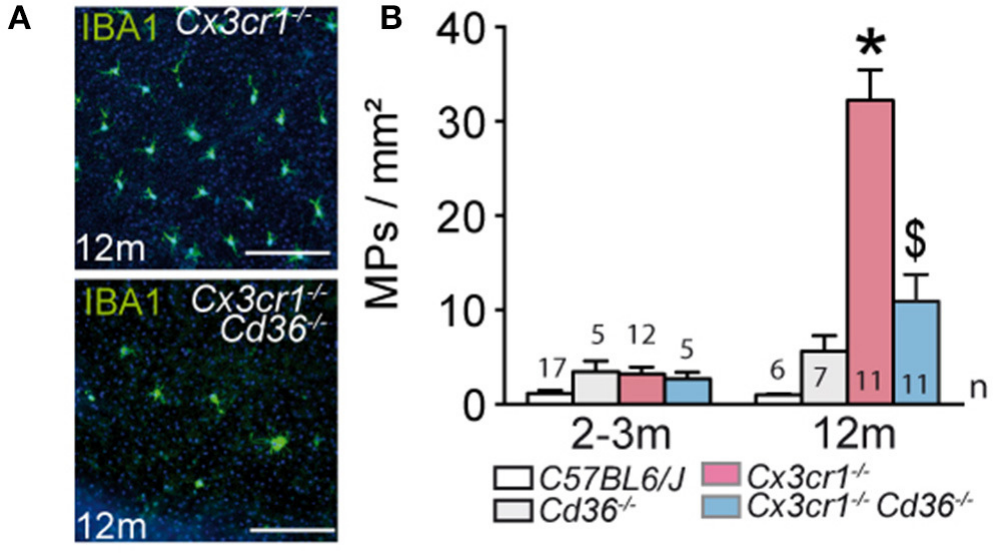
CD36 deficiency prevents age-related chronic subretinal inflammation in Cx3cr1-knockout mice. **(A)** Representative images of 12 month old IBA-1 (green) stained RPE flatmounts of 12 months old *Cx3cr1*^−/−^ and *Cx3cr1*^−/−^*Cd36*^−/−^ mice. **(B)** Quantification of subretinal IBA-1+MPs in 2-3 months (left) and 12 months (right) old mice of the indicated strains (*Mann & Whitney test at 12 months of *Cx3cr1*^−/−^ vs. 3 months old *Cx3cr1*^−/−^ mice *p* = 0.0002; ^$^Mann & Whitney test at 12 months of *Cx3cr1*^−/−^ vs. 12 months old *Cx3cr1*^−/−^*Cd36*^−/−^ mice *p* < 0.0001). *n* = number of replicates indicated in the graphs; replicates represent quantification of eyes from different mice of at least three different cages. Scale bar **(A)** = 50 m.

**Figure 2:**
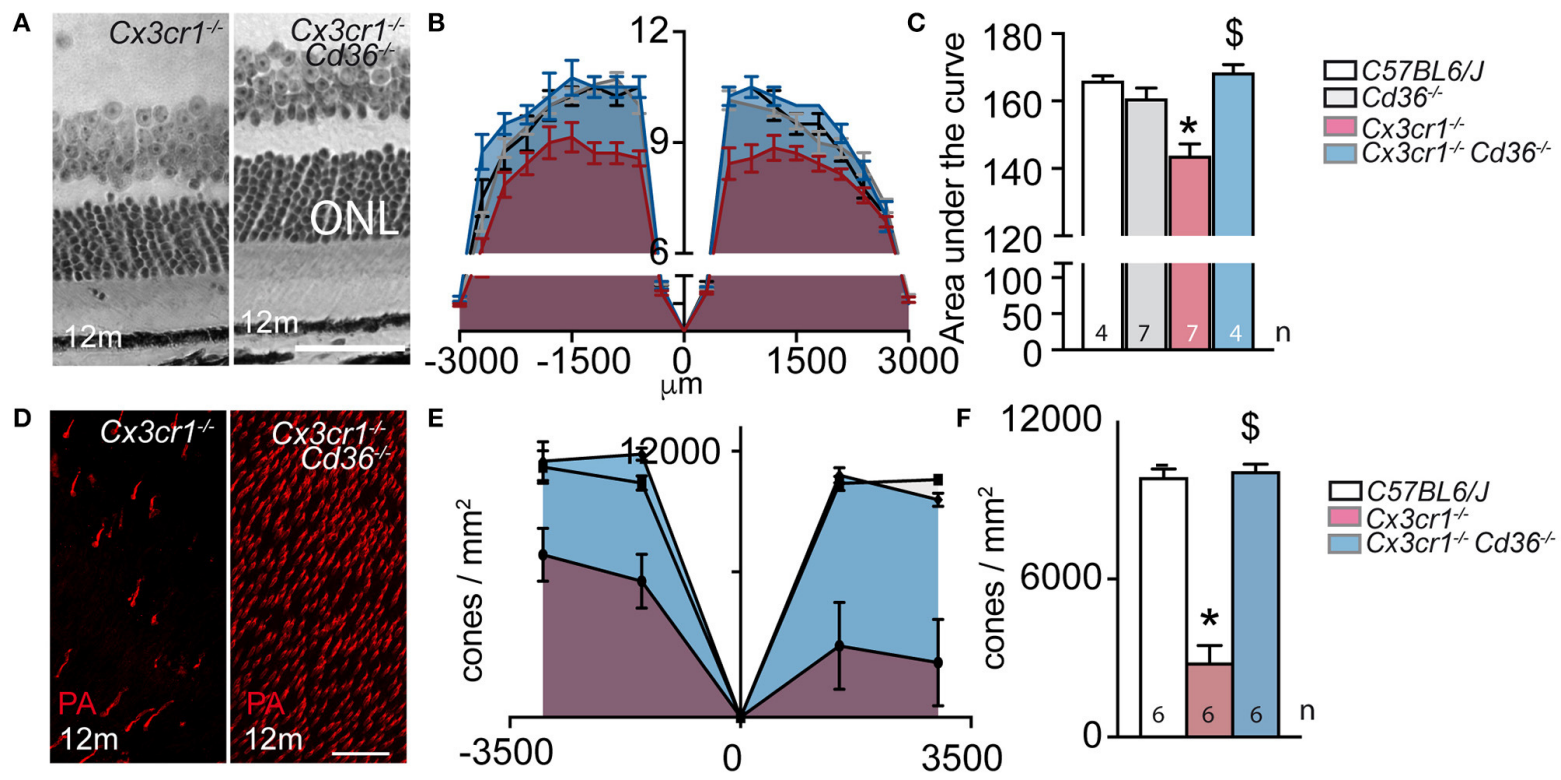
CD36 deficiency prevents age-related rod and cone degeneration in Cx3cr1-knockout mice. **(A)** Micrographs, taken 1,000 μm from the optic nerve, of 12 month-old *Cx3cr1*^−/−^ and *Cx3cr1*^−/−^*Cd36*^−/−^ mice. **(B)** Photoreceptor nuclei rows at increasing distances (−3,000 μm: inferior pole, +3,000 μm: superior pole) from the optic nerve (0 μm) in 12 month-old mice. **(C)** Quantification of the area under the curve of photoreceptor nuclei row counts of 12 month-old mice of the indicated transgenic mouse strains (Mann Whitney wildtype vs. *Cx3cr1*^−/−^ mice *p = 0.0024; Mann Whitney *Cx3cr1*^−/−^ vs. *Cx3cr1*^−/−^*Cd36*^−/−^ mice ^$^*p* = 0.0024). **(D)** Micrographs, taken in the superior periphery of peanut agglutinin (staining cone segments, red) stained 12 month-old *Cx3cr1*^−/−^ and *Cx3cr1*^−/−^*Cd36*^−/−^ mice. **(E)** Cone density quantification on retinal flatmounts in peripheral and central, inferior and superior retina (−3,000 μm: inferior pole, +3,000 μm: superior pole, optic nerve: 0 μm) and their average **(F)** in 12 month-old mice of the indicated mouse strains (Mann Whitney wildtype vs. *Cx3cr1*^−/−^ mice *p = 0.0022; Mann Whitney *Cx3cr1*^−/−^ vs. *Cx3cr1*^−/−^*Cd36*^−/−^ mice ^$^*p* = 0.0022). ONL, outer nuclear layer; PNA, peanut agglutinin Scale bar **(A,D)** = 50 μm. *n* = number of replicates indicated in the graphs; replicates represent quantification of eyes from different mice of at least three different cages.

We first sought to determine whether *Cd36* deficiency would alter disease onset and progression in *Cx3cr1*^−/−^ mice. Quantification of subretinal IBA-1^+^ MPs on retinal and RPE/choroidal flatmounts of 2–3 month and 12 month old animals showed that the age-related increase in subretinal MPs observed in *Cx3cr1*^−/−^ mice was significantly inhibited in the absence of CD36 (Figures 1A,B). Next, we evaluated whether *Cd36* deficiency influenced the rod and cone degeneration that is associated with MP accumulation in *Cx3cr1*^−/−^ mice (5, 6, 29). Micrographs revealed that *Cx3cr1*^−/−^*Cd36*^−/−^ mice were protected against the thinning of the outer nuclear layer that hosts the photoreceptor nuclei, which is observed in aged *Cx3cr1*-deficient mice (6) (Figure 2A). Photoreceptor nuclei row counts (Figure 2B) and calculation of the area under the curve showed *CD36* deficiency protected against the photoreceptor cell loss observed in *Cx3cr1*^−/−^ mice (Figure 2C), while only a slight decrease was observed in *CD36*^−/−^ compared to control-mice. Similarly, *CD36* deficiency completely protected against cone loss observed on peanut agglutinin stained retinal flatmounts of 12 month-old *Cx3cr1*^−/−^ mice (Figures 2D–F). Thus, we show that CD36 contributes importantly to the chronic, age-related subretinal MP accumulation and associated photoreceptor degeneration observed in *Cx3cr1*^−/−^ mice.

### CD36 Deficiency Prevents Pathogenic Subretinal Inflammation in Acute, Light-Induced Cx3cr1-Knockout Mice

**Figure 3:**
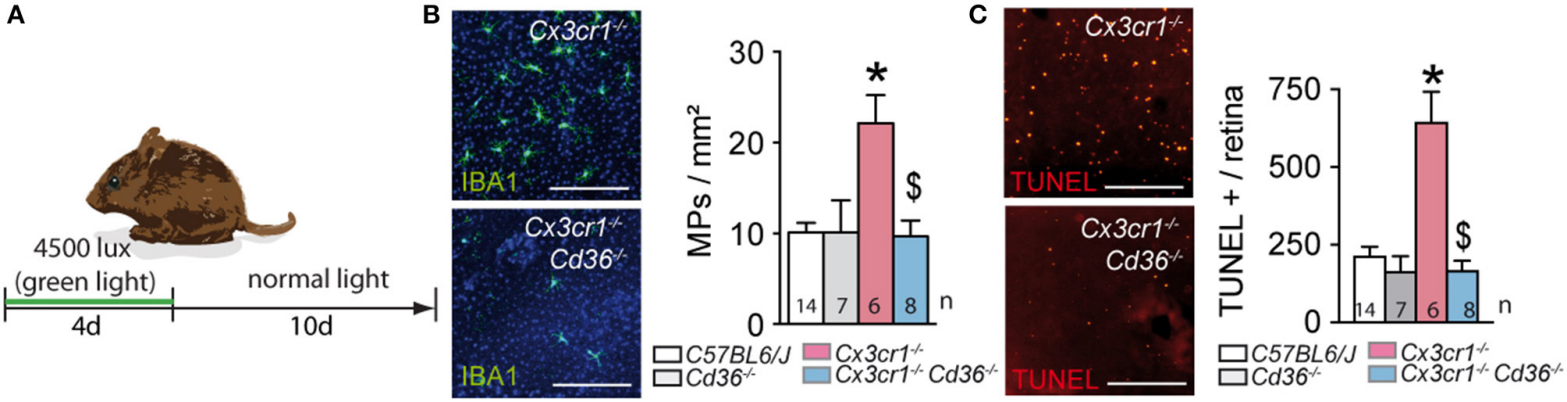
CD36 deficiency prevents pathogenic subretinal inflammation in acute, light-induced Cx3cr1-knockout mice. **(A)** Schematic representation of the light-challenge model. **(B)** Representative images and quantification of IBA-1 (green) stained RPE flatmounts of 2 month-old mice of the indicated strains after 10 days after the light-challenge (*Mann & Whitney test C57BL/6J control mice vs. *Cx3cr1*^−/−^ mice *p* = 0.0014; ^$^Mann & Whitney test *Cx3cr1*^−/−^ vs. *Cx3cr1*^−/−^*Cd36*^−/−^ mice *p* = 0.02). **(C)** Representative images and quantification of TUNEL+ cells in the outer nuclear layer of TUNEL-stained retinal flatmounts of d14 light-challenged mice of the indicated strains (*Mann & Whitney test C57BL/6J control mice vs. *Cx3cr1*^−/−^ mice *p* = 0.0005; ^$^Mann & Whitney test *Cx3cr1*^−/−^ vs. *Cx3cr1*^−/−^*Cd36*^−/−^ mice *p* = 0.0012). *n*, indicated for each bar; Scale bar **(A)** = 50 μm.

Next, we evaluated its impact on acute light-induced stress. The intensity of the light-challenge model used herein was calibrated to induce substantial subretinal MP infiltration in AMD-prone *Cx3cr1*^−/−^-mice but not in *C57BL6/J* controls (6). Quantification of subretinal IBA-1^+^ MPs on retinal and RPE/choroidal flatmounts of 2–3 month old animals that were exposed to 4500 lux of green light with fully dilated pupils for 96 h, followed by 10 days of housing in the normal animal quarters, revealed an increase in subretinal MPs in *Cx3cr1*^−/−^ mice, which was significantly inhibited in the absence of CD36 (Figures 3A,B). To quantify photoreceptor apoptosis in light-challenged mice we prepared TUNEL stained retinal flatmounts as previously described (5, 18). Flatmounts from *Cx3cr1*^−/−^mice displayed TUNEL positive cells in the outer nuclear layer, where the photoreceptor nuclei are located, to a much greater extent than *C57BL6/J* controls as previously reported (6). Importantly, *Cx3cr1*^−/−^*Cd36*^−/−^ mice were protected against the photoreceptor apoptosis observed in Cx3cr1^−/–^mice (Figure 3C).

Taken together, CD36-deficiency very significantly inhibited the acute subretinal inflammation and associated photoreceptor apoptosis induced in the light-challenged *Cx3cr1*^−/−^mice, similar to our observations in chronic age-related inflammation.

### CD36 Deficiency Normalizes the Resistance to Subretinal Elimination of Cx3cr1-Deficient Monocytes and Reduces Their IL-6 Production *in vitro*

We have previously shown that subretinal MPs in aged- and light-challenges *Cx3cr1*^−/−^ mice are in part derived from infiltrating Mo (6). We showed that subretinally injected WT Mos, MCs or Mφs quickly undergo apoptosis and are eliminated (4), and that such clearance was significantly delayed when MPs lacked CX3CR1 (4). We demonstrated that this resistance was at least in part due to the fact that Cx3cr1-deficient MPs overexpress interleukin 6 (IL-6), which downregulates FasL expression the RPE that normally induces subretinal MP elimination (4).

**Figure 4:**
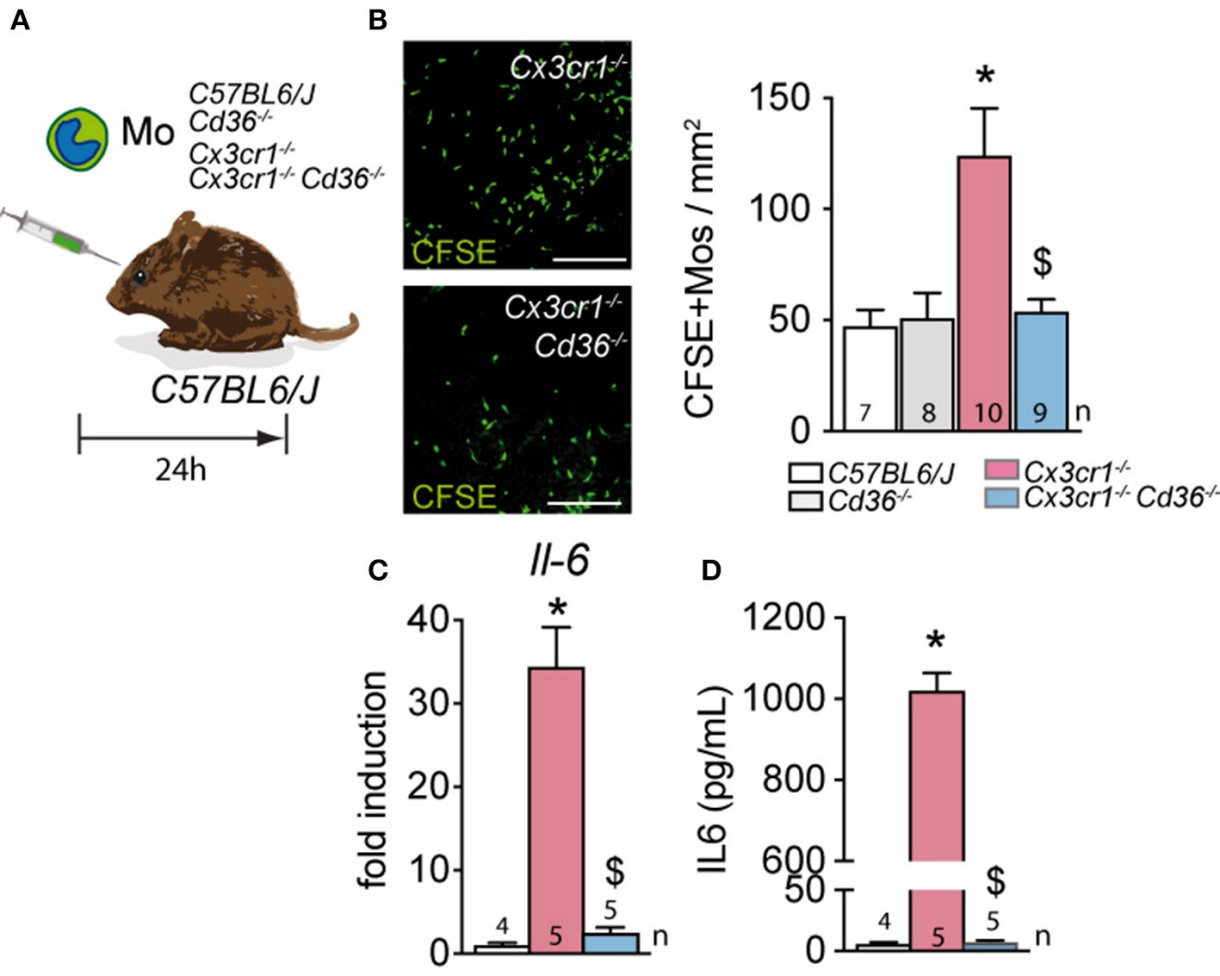
CD36 deficiency normalizes the resistance to subretinal elimination of Cx3cr1-deficient monocytes and reduces their IL-6 production *in vitro*. **(A)** Schematic representation of the subretinal adoptive transfer experiments. **(B)** Representative images and quantification of CSFE+ Mo of the indicated strains on RPE flatmounts 24 h after subretinal transfer to the subretinal space of wildtype mice (*Mann & Whitney test C57BL/6J Mo vs. *Cx3cr1*^−/−^ Mo *p* = 0.0019; ^$^Mann & Whitney test *Cx3cr1*^−/−^ vs. *Cx3cr1*^−/−^*Cd36*^−/−^ Mos *p* = 0.0079). **(C)** Quantitative RT–PCR of IL-6 mRNA normalized with S26 mRNA of C57BL/6J, *Cx3cr1*^−/−^, and *Cx3cr1*^−/−^*Cd36*^−/−^ monocytes cultured for 24 h (*Mann & Whitney test C57BL/6J Mo vs. *Cx3cr1*^−/−^ Mo *p* = 0.0159; ^$^Mann & Whitney test *Cx3cr1*^−/−^ vs. *Cx3cr1*^−/−^*Cd36*^−/−^ Mos *p* = 0.0159). **(D)** IL-6 ELISA of the supernatants of C57BL/6J, *Cx3cr1*^−/−^-, and *Cx3cr1*^−/−^*Cd36*^−/−^ monocytes cultured for 24 h (*Mann & Whitney test C57BL/6J Mo vs. *Cx3cr1*^−/−^ Mo *p* = 0.0159; ^$^Mann & Whitney test *Cx3cr1*^−/−^ vs. *Cx3cr1*^−/−^*Cd36*^−/−^ Mos *p* = 0.0079). *n*, indicated for each bar; Scale bar **(A)** = 50 μm.

To evaluate whether CD36 expression on MPs influences their susceptibility to be eliminated by the RPE, we adoptively transferred CFSE-labeled Mos from the different mouse strains into the subretinal space of wildtype mice. The surviving CFSE^+^ Mos were then enumerated 24 h after injection (Figure 4A). As previously shown, *Cx3cr1*-deficient Mo were significantly more numerous than subretinally injected wildtype Mos. Importantly, the deletion of *Cd36* in *Cx3cr1*-deficient Mo completely reversed this effect (Figure 4B).

*Cx3cr1*-deficient MPs express increased levels of APOE, which we showed activates the TLR2-CD14-dependent innate immunity receptor cluster and induces IL-6 (4). CD36 is a co-receptor of the toll-like receptor 2 (TLR2) and involved in the proinflammatory signaling cascade and the release of inflammatory cytokines (41, 42). Indeed, IL-6 transcription measured by RT-PCR (Figure 4C) and IL-6 secretion evaluated by ELISA (Figure 4D) was significantly increased in bone marrow derived Mo from *Cx3cr1*-deficient mice compared to control, but the deletion of *Cd36* in *Cx3cr1*-deficient Mo prevented this increase of expression.

Taken together this data demonstrates that *Cd36* deletion in *Cx3cr1*-deficient Mos inhibits the over-expression of IL-6 in *Cx3cr1*-deficient Mos and reverses their ability to withstand the immunosuppressive environment of the subretinal space.

## Discussion

We previously showed that the deletion of *Cx3cr1*, a gene that is exclusively expressed on MPs in the adult eye, is sufficient to trigger an age-related or light-induced pathogenic non-resolving subretinal inflammation. We here demonstrate that the deletion of CD36 in *Cx3cr1*-deficient mice prevented the age- and light-challenge-dependent accumulation of subretinal MPs and protected against the associated photoreceptor cell death. Our data demonstrates that CD36 expression on Mo is necessary for the over-expression of IL-6 that we observe in *Cx3cr1*-deficient MdCs. We previously showed that IL-6 expression in *Cx3cr1*-deficient MPs is necessary for the accumulation of subretinal MPs in *Cx3cr1*-deficient mice, as it induces the downregulation of FasL expression the RPE that induces subretinal MP elimination in wildtype animals (4). Indeed, when we subretinally adoptively transferred *Cx3cr1*^−/−^- and *Cd36*^−/−^*Cx3cr1*^−/−^-Mo to wildtype recipients, the deletion of *Cd36* on *Cx3cr1*^−/−^-monocytes restored the susceptible to subretinal elimination to the level observed in wildtype monocytes. Taken together our data demonstrates that Cd36 deficiency inhibits retinal inflammation and retinal degeneration in Cx3cr1 knockout mice.

We previously showed that *Cx3cr1*-deficient MPs are characterized by an over-expression of APOE that triggers NFκB activation and cytokine secretion. *ApoE*-deletion in *Cx3cr1*-deficient mice significantly prevents the accumulation of pathogenic subretinal MPs (4, 6, 27), similar to *Cd36* deficiency. Mechanistically, we showed that the excess of APOE activates the CD14/TLR2-dependent innate immunity receptor complex (IIRC) on MPs (4, 27). This is likely due to APOE-induced cholesterol extraction from the lipid rafts of MPs, which lifts the physiological separation of CD14 (located in the lipid raft) from TLR2 (located in non-lipid raft membrane) and triggers NFκB activation and cytokine secretion in the absence of TLR ligands, as previously demonstrated for APOA-I (28, 46).

CD36 is also part of the innate immunity receptor complex (47, 48) and an obligate co-receptor of the toll-like receptor 2 (TLR2). Its inhibition blocks the TLR2-dependent NFκB activation and pro-inflammatory signaling cascade and the release of inflammatory cytokines such as IL-6 (41, 42). In *Cx3cr1*-deficient mice the deletion of *Cd36* therefore likely counters the APOE-induced activation of the IIRC, activation of NFκB and IL-6 secretion, which we show reduces RPE FasL expression and MP elimination (4). As a result, the CD36-deficient MPs are quickly eliminated from the subretinal space, preventing pathogenic subretinal inflammation. Interestingly, it has recently been demonstrated that *Cd36* deletion and pharmacological inhibition of CD36 also inhibits pathogenic inflammation in an acute model of light-induced degeneration in *Cx3cr1*-competent mice (42). The authors demonstrated that the protective effect is likely due to the inhibition of IL-1 that we showed also plays an important role in photoreceptor degeneration in *Cx3cr1*-deficient mice (3, 18).

In summary, the inhibition of CD36 on MPs in diseases that are characterized by pathogenic subretinal inflammation such as AMD, holds the promise to reduce MP accumulation and their production of pathogenic cytokines. On the other hand CD36 expressed by RPE cells has an important role in the maintenance of the choriocapillaries and the elimination of lipids from Bruchs membrane (39, 45). Ideally, future therapies for AMD might therefore want to specifically target CD36 on MPs.

## Materials and Methods

### Animals

*Cx3cr1*^−/−^*CD36*^−/−^mouse strains on C57BL/6 background were generated from *Cx3cr1*^−/−^mice and *CD36*^−/−^mouse strains, which were generated as previously described (49, 50). All mice were negative for the *Crb1*^*rd*8^, *Pde6b*^*rd*1^, and *Gnat2*^*cpfl*3^ mutations. Mice were housed in the animal facility under specific pathogen-free condition, in a 12/12 h light/dark (100–500 lux) cycle with water and normal diet food available *ad libitum*. All experimental protocols and procedures were approved by the local animal care ethics committee “Comité d'Éthique en Expérimentation Animale Charles Darwin” (N° p3/2008/54).

### Light-Challenge Model

Two- to four-month-old mice were adapted to darkness for 6 h and pupils were fully dilated with 1% Atropine (Novartis). Animals were then exposed to green LED light (4500 Lux, JP Vezon équipements) for 4 days and subsequently kept in cyclic 12/12 h normal animal facility conditions. MP accumulation and retinal degeneration were assessed at 10 days after light exposure (6).

### Immunohistochemistry and TUNEL Staining of Retinal Flatmounts

Eyes were enucleated, fixed in 4% paraformaldehyde for 20 min at room temperature and sectioned at the limbus; the cornea and lens were discarded. The retinas were peeled from the RPE/choroid/sclera and incubated overnight at 4°C in PBS-1% triton with the following primary antibodies: peanut agglutinin Alexa fluor® 594 (Thermo Fisher Scientific; 1/100), and goat polyclonal anti-IBA1 (1/100, Wako). After few washes, the retinas were incubated for 2 h at room temperature with appropriate Alexa Fluor® conjugated secondary antibodies (Thermo Fisher Scientific; 1:500) in PBS-1% triton and nuclei were counterstained with Hoechst (1:1,000, Sigma Aldrich). The retinas were flatmounted and viewed with a fluorescence microscope (DM5500, Leica). Images centered on the area with the lowest number of PNA+ cone arrestin+ cells were captured with a confocal laser-scanning microscope (FV1000, Olympus) using a 40X lens. Each cell population was manually counted in a masked fashion. IBA-1+ cells were quantified on flatmounts on the outer segment side of the detached retina while PNA+ cone arrestin+ cells were counted on confocal microscopy Z-stacks using ImageJ software.

For histology, eyes were fixed in 0.5% glutaraldehyde, 4% PFA for 2 h, dehydrated and mounted in Historesin (Leica). Five millimeters of oriented sections crossing inferior pole, optic nerve and superior pole were cut and stained with toluidin blue. Rows of nuclei in the ONL were counted at different distances from the optic nerve (6).

For Terminal deoxynucleotidyl transferase dUTP nick end labeling (TUNEL), 4% PFA fixed retinal flatmounts were pre-treated with frozen methanol for 30 min and then frozen methanol/acetic acid (2:1) for another 30 min. After washing with PBS, flatmounts were incubated overnight at 4°C with the reaction mixture as described by manufacturer's protocol (*In Situ* Cell Death Detection Kit, Roche Diagnostics) and then for 90 min at 37°C. After reaction was stopped by washing with PBS at RT, nuclei were counterstained with Hoechst (Sigma–Aldrich).

### Cell Preparations and Cell Culture

Bone marrow-derived monocytes (in serum-free X-Vivo 15 medium) were performed as previously described (6). RT-PCRs using Sybr Green (Life Technologies) and ELISAs using mouse IL-6 DuoSet (R&D Systems) were performed as previously described (6).

### Subretinal Mononuclear Phagocyte Cell Clearance

Bone marrow-derived monocytes (~95% pure) were labeled in 10 μM CFSE (Life technologies). Cells were washed and resuspended in PBS. Twelve thousand cells (4 μl) were injected in the subretinal space of anesthetized 2 month-old mice using a microinjector and glass microcapillaries (Eppendorf). A hole was pierced with the glass capillary prior to the subretinal injection to avoid intra-ocular pressure increase and to allow retinal detachment with 4 μl of solution. The subretinal injection was verified by fundoscopy. Eyes were enucleated after 24 h, fixed in 4% PFA, and flatmounted. CFSE^+^transplanted cells were counted on the subretinal aspect of the retinal flatmount and the RPE/choroid flatmount of each eye. Eyes with subretinal hemorrhages were discarded.

### Statistical Analysis

Sample sizes for our experiments were determined according to our previous studies Graph Pad Prism 6 (GraphPad Software) was used for data analysis and graphic representation. All values are reported as mean ± SEM. Statistical analysis was performed by Mann–Whitney *U*-test (2-group comparisons). The *n* and *p*-values are indicated in the figure legends.

## Data Availability Statement

All datasets generated for this study are included in the article.

## Ethics Statement

The animal study was reviewed and approved by Comité d'Ethique en Expérimentation Animale Charles Darwin.

## Author Contributions

SL, J-BC, ST, CR, MH, SA, and WR conducted the experiments. CC, MF, HO, SC, J-AS, CD, XG, and FS designed and analyzed the experiments.

### Conflict of Interest

The authors declare that the research was conducted in the absence of any commercial or financial relationships that could be construed as a potential conflict of interest.
